# Atrial Flutter Secondary to Severe Laxative-Induced Hypomagnesemia in Anorexia Nervosa: A Case Report

**DOI:** 10.7759/cureus.114337

**Published:** 2026-08-11

**Authors:** Srihita Patibandla, Keshavkiran Jayagopi, Ahmed El-Aawar, Darab Shuja, Ali Z Ansari, Zayn I Haque, Sahar Hafeez

**Affiliations:** 1 Department of Internal Medicine, Trinity Health Grand Rapids Hospital, Grand Rapids, USA; 2 Department of Cardiology, Merit Health Wesley, Hattiesburg, USA; 3 Department of Internal Medicine, William Carey University College of Osteopathic Medicine, Hattiesburg, USA; 4 Department of Family Medicine, William Carey University College of Osteopathic Medicine, Hattiesburg, USA; 5 Department of Internal Medicine/Cardiology, Merit Health Wesley, Hattiesburg, USA; 6 Department of Family Medicine, Prisma Health Tuomey Hospital, Sumter, USA; 7 Department of Family Medicine, University of South Carolina Floyd School of Medicine, Columbia, USA; 8 Department of Internal Medicine, Lincoln Memorial University-DeBusk College of Osteopathic Medicine, Harrogate, USA; 9 Department of Pathology and Laboratory Medicine, William Carey University College of Osteopathic Medicine, Hattiesburg, USA

**Keywords:** anorexia nervosa, atrial flutter, eating disorders, electrolyte imbalance, generalized anxiety disorder, hypomagnesemia, laxative abuse, malnutrition, purging behavior, reversible arrhythmia

## Abstract

Atrial flutter is an uncommon supraventricular arrhythmia in young adults without structural heart disease and is typically associated with underlying cardiac pathology rather than isolated metabolic abnormalities. Electrolyte disturbances, particularly hypomagnesemia, can contribute to myocardial electrical instability, but they rarely present as the primary cause of atrial flutter in otherwise healthy young individuals. We present the case of a 23-year-old woman with a history of anorexia nervosa and generalized anxiety disorder who presented with acute palpitations, dizziness, and near-syncope. An electrocardiogram (ECG) was performed and demonstrated typical atrial flutter with a 4:1 atrioventricular conduction pattern in the setting of profound hypomagnesemia secondary to chronic stimulant laxative abuse. Laboratory studies also revealed mild hypokalemia and metabolic alkalosis consistent with ongoing gastrointestinal losses and severe nutritional restriction. She was treated with intravenous (IV) magnesium sulfate and potassium replacement without the use of antiarrhythmic medications, rate-control agents, or electrical cardioversion. Following correction of her electrolyte abnormalities, she spontaneously converted to normal sinus rhythm within several hours, with complete resolution of symptoms and no recurrence during her hospital stay. This case highlights how severe hypomagnesemia may have been a significant contributing factor to atrial flutter in a young patient without structural heart disease. It also emphasizes the importance of promptly identifying and correcting electrolyte abnormalities in patients with eating disorders and laxative abuse, as early treatment can rapidly reverse potentially serious but reversible cardiac manifestations and avoid unnecessary invasive management.

## Introduction

Atrial flutter is a macroreentrant supraventricular tachyarrhythmia characterized by rapid and organized atrial depolarization, most commonly with atrial rates ranging from 250 to 300 beats per minute [1]. It is typically encountered in older adult populations and is most often associated with structural heart disease, including heart failure secondary to coronary artery disease, valvular heart disease, chronic obstructive pulmonary disease, or postoperative cardiac states [2]. In these settings, atrial remodeling and fibrosis create a substrate for reentrant electrical circuits, most commonly involving the cavotricuspid isthmus [3]. In contrast, atrial flutter in young individuals without known structural cardiac abnormalities is relatively rare and often warrants evaluation for secondary and reversible etiologies [4]. These may include acute inflammatory conditions such as myocarditis, exposure to stimulants or sympathomimetic agents, endocrine abnormalities such as thyrotoxicosis, congenital conduction system abnormalities, or significant metabolic disturbances that alter myocardial excitability and conduction velocity [2,4]. In young patients presenting with atrial flutter, evaluation for these potentially reversible secondary causes is therefore important, particularly when structural heart disease is not evident.

Among metabolic causes, electrolyte abnormalities play a particularly important role in the development of cardiac arrhythmias due to their direct effects on myocardial cell membrane stability and ion channel function [5]. Magnesium is a critical cofactor in numerous cellular processes involved in cardiac electrophysiology, including regulation of sodium-potassium ATPase activity, modulation of calcium influx through L-type calcium channels, and stabilization of myocardial resting membrane potential [6]. Severe hypomagnesemia can increase myocardial excitability, shorten atrial refractory periods, and promote the development of both atrial and ventricular arrhythmias, particularly in the presence of concomitant hypokalemia [7]. Potassium depletion further exacerbates electrical instability by impairing repolarization and increasing susceptibility to reentrant rhythms [8]. These disturbances are often seen in conditions associated with chronic gastrointestinal losses, malnutrition, or renal wasting [2,4]. One clinically important but sometimes underrecognized source of such losses is chronic stimulant laxative use, which can result in persistent fluid depletion, metabolic alkalosis, and significant depletion of key electrolytes [9]. This pattern is particularly relevant in patients with eating disorders such as anorexia nervosa, where laxative abuse may be used as a maladaptive weight-control behavior and can contribute to profound physiologic and cardiac consequences over time [4,9].

## Case presentation

A 23-year-old woman with a past medical history significant for anorexia nervosa and generalized anxiety disorder presented to the emergency department with complaints of persistent palpitations, dizziness, generalized weakness, and two episodes of near-syncope that began approximately six hours prior to presentation. She described the sensation as a sudden onset of a rapid, irregular “fluttering” sensation in her chest associated with intermittent lightheadedness and episodes of blurred vision, particularly pronounced with positional changes and attempts to stand or ambulate. Although she denied complete loss of consciousness, she reported that the presyncopal symptoms were severe enough that she had to intentionally lower herself to the ground on two separate occasions to avoid falling. She additionally endorsed mild exertional dyspnea with minimal activity, profound fatigue that limited her usual daily functioning, and intermittent non-radiating chest discomfort described as a vague substernal tightness without a clear pleuritic or exertional component. She denied fever, chills, cough, recent upper respiratory illness, nausea, vomiting, diarrhea, illicit drug use, alcohol consumption, excessive caffeine intake, energy drink use, or any recent exposure to sympathomimetic or stimulant medications.

The patient reported that her symptoms had developed insidiously over the preceding week, initially manifesting as intermittent brief episodes of palpitations associated with fatigue that would self-resolve without intervention. Over time, these episodes became more frequent and progressively more intense, culminating in a sustained episode of palpitations on the day of presentation that did not resolve spontaneously and was accompanied by worsening dizziness, generalized weakness, and reduced exercise tolerance. She described feeling increasingly “unsteady” and noted difficulty performing routine activities due to fatigue and intermittent lightheadedness. The persistence and escalation of symptoms ultimately prompted her to seek evaluation in the emergency department. She denied any previous history of known cardiac disease, congenital heart defects, prior arrhythmias, hypertension, diabetes mellitus, thyroid disease, or prior hospital admissions for cardiovascular complaints. There was no known family history of sudden cardiac death, inherited channelopathies, cardiomyopathy, or premature coronary artery disease, and she was not aware of any familial history of unexplained syncope or arrhythmic disorders.

Further history revealed a longstanding diagnosis of anorexia nervosa characterized by severe restrictive eating behaviors, persistent body image distortion, and an intense and pervasive fear of weight gain. Over the preceding year, she reported progressive and unintentional weight loss of approximately 16 kilograms, with a sustained daily caloric intake estimated between 500 and 700 kilocalories despite ongoing nutritional counseling in the past. She frequently skipped meals, avoided calorie-dense foods, and maintained rigid dietary restrictions, often expressing significant distress when discussing food intake or weight changes. She described persistent preoccupation with body shape and caloric restriction, with increased rigidity in eating behaviors during periods of psychological stress. During a more detailed interview, she disclosed chronic use of over-the-counter stimulant laxatives, specifically bisacodyl (Dulcolax), as a compensatory weight-control behavior, admitting to ingesting approximately 10 to 15 tablets daily over the preceding eight months. She stated that her laxative use had gradually escalated over time due to a belief that it prevented weight gain and “cleared” ingested calories, despite recognizing that it led to intermittent abdominal cramping, urgency, and loose stools. She denied self-induced vomiting but acknowledged engaging in excessive physical activity several times per week, often exercising despite significant fatigue and reduced nutritional intake, further contributing to her overall physiologic depletion.

Her psychiatric history was notable for generalized anxiety disorder diagnosed during adolescence, for which she had been prescribed sertraline 50 mg daily with partial prior benefit. She acknowledged inconsistent adherence to her medication regimen over the preceding several months, citing concerns that pharmacologic treatment could contribute to weight gain and negatively impact her body image goals. She described worsening baseline anxiety related to academic demands, interpersonal stressors, and health-related fears, which in turn appeared to exacerbate her restrictive eating behaviors and increase reliance on laxative use as a maladaptive coping mechanism. She also reported that periods of heightened anxiety were closely associated with more severe dietary restriction and increased compulsive behaviors related to weight control, suggesting a cyclical interaction between psychiatric symptoms and disordered eating patterns.

Upon arrival to the emergency department, the patient appeared frail, cachectic, and visibly anxious but remained alert, cooperative, and oriented to person, place, time, and situation. She demonstrated a markedly thin body habitus with prominent temporal muscle wasting, loss of buccal fat pads, and clearly diminished subcutaneous fat stores throughout the upper and lower extremities. Her skin appeared dry and cool to touch, and mucous membranes were notably dry, consistent with mild volume depletion. Hair was brittle with mild diffuse alopecia, further supporting chronic nutritional deficiency. Initial vital signs demonstrated a temperature of 36.5°C, blood pressure of 98/62 mmHg, heart rate of 61 beats per minute, respiratory rate of 18 breaths per minute, and oxygen saturation of 99% on room air. Although hemodynamically stable at presentation, her blood pressure was borderline low, consistent with possible chronic undernutrition and reduced intravascular volume. Her height was 165 cm, and weight was 42 kg, corresponding to a body mass index of 15.4 kg/m^2^, which is consistent with severe undernutrition and medically significant malnutrition.

Cardiovascular examination revealed a regular rhythm on auscultation despite the patient’s subjective sensation of ongoing palpitations at the time of evaluation. No murmurs, rubs, or gallops were appreciated, and there was no clinical evidence of structural valvular disease on bedside examination. Peripheral pulses were palpable, symmetric, and slightly diminished in amplitude but without delay or asymmetry. There was no peripheral edema, and jugular venous pressure was not elevated, suggesting absence of overt volume overload or heart failure physiology. Pulmonary examination demonstrated clear and equal breath sounds bilaterally with no wheezing, rales, or crackles, and no signs of respiratory distress were observed. Abdominal examination revealed a soft, nondistended abdomen with mild diffuse tenderness to deep palpation but no rebound tenderness, guarding, or palpable masses, and no hepatosplenomegaly was appreciated. Bowel sounds were present and normoactive in all quadrants. Neurological examination demonstrated intact cranial nerves II through XII, preserved motor strength in all extremities, normal gross sensation, and no focal neurological deficits or signs of acute encephalopathy. Psychiatric assessment revealed a visibly anxious affect with intermittent psychomotor restlessness, poor eye contact, and marked preoccupation with body weight and nutritional status. She demonstrated limited insight into the severity of her malnutrition and its potential medical consequences, despite being able to answer questions appropriately and participate in the examination.

Initial laboratory evaluation demonstrated multiple significant electrolyte abnormalities consistent with chronic gastrointestinal and volume-depletion-related losses (Table 1). The most striking abnormality was profound hypomagnesemia, with a serum magnesium concentration of 0.8 mg/dL, well below the normal physiologic range and consistent with severe total-body magnesium depletion. Mild hypokalemia was also present, along with borderline hypocalcemia, both of which are commonly seen in the setting of chronic magnesium deficiency due to its role in renal potassium handling and parathyroid hormone-mediated calcium regulation. Serum bicarbonate was elevated, suggestive of a chronic metabolic alkalosis pattern, most consistent with ongoing volume contraction and gastrointestinal electrolyte losses in the context of prolonged laxative use. Despite these abnormalities, renal function was preserved with a normal blood urea nitrogen and creatinine, indicating an absence of intrinsic renal injury or acute kidney dysfunction. Cardiac biomarkers, including high-sensitivity troponin I, showed no evidence of myocardial injury or ischemia. Thyroid function testing was within normal limits, effectively reducing the likelihood of endocrine-mediated arrhythmogenesis such as thyrotoxicosis contributing to her clinical presentation.

**Table 1:** Initial laboratory evaluation on presentation demonstrating significant electrolyte abnormalities, including profound hypomagnesemia and mild hypokalemia.

Laboratory Test	Result	Reference Range
Sodium	137 mmol/L	135-145 mmol/L
Potassium	3.1 mmol/L	3.5-5.0 mmol/L
Chloride	94 mmol/L	98-107 mmol/L
Bicarbonate	31 mmol/L	22-29 mmol/L
Magnesium	0.8 mg/dL	1.7-2.4 mg/dL
Calcium	8.4 mg/dL	8.5-10.5 mg/dL
Phosphorus	2.6 mg/dL	2.5-4.5 mg/dL
Blood urea nitrogen	15 mg/dL	7-20 mg/dL
Creatinine	0.58 mg/dL	0.6-1.2 mg/dL
Glucose	82 mg/dL	70-99 mg/dL
Aspartate aminotransferase	24 U/L	10-40 U/L
Alanine aminotransferase	18 U/L	7-56 U/L
Albumin	3.6 g/dL	3.5-5.0 g/dL
White blood cell count	5.9 ×10^9^/L	4.0-11.0 ×10^9^/L
Hemoglobin	11.7 g/dL	12.0-16.0 g/dL
Platelet count	262 ×10^9^/L	150-400 ×10^9^/L
High-sensitivity troponin I	<5 ng/L	<14 ng/L
Thyroid-stimulating hormone	2.04 μIU/mL	0.4-4.5 μIU/mL

**Figure 1:**
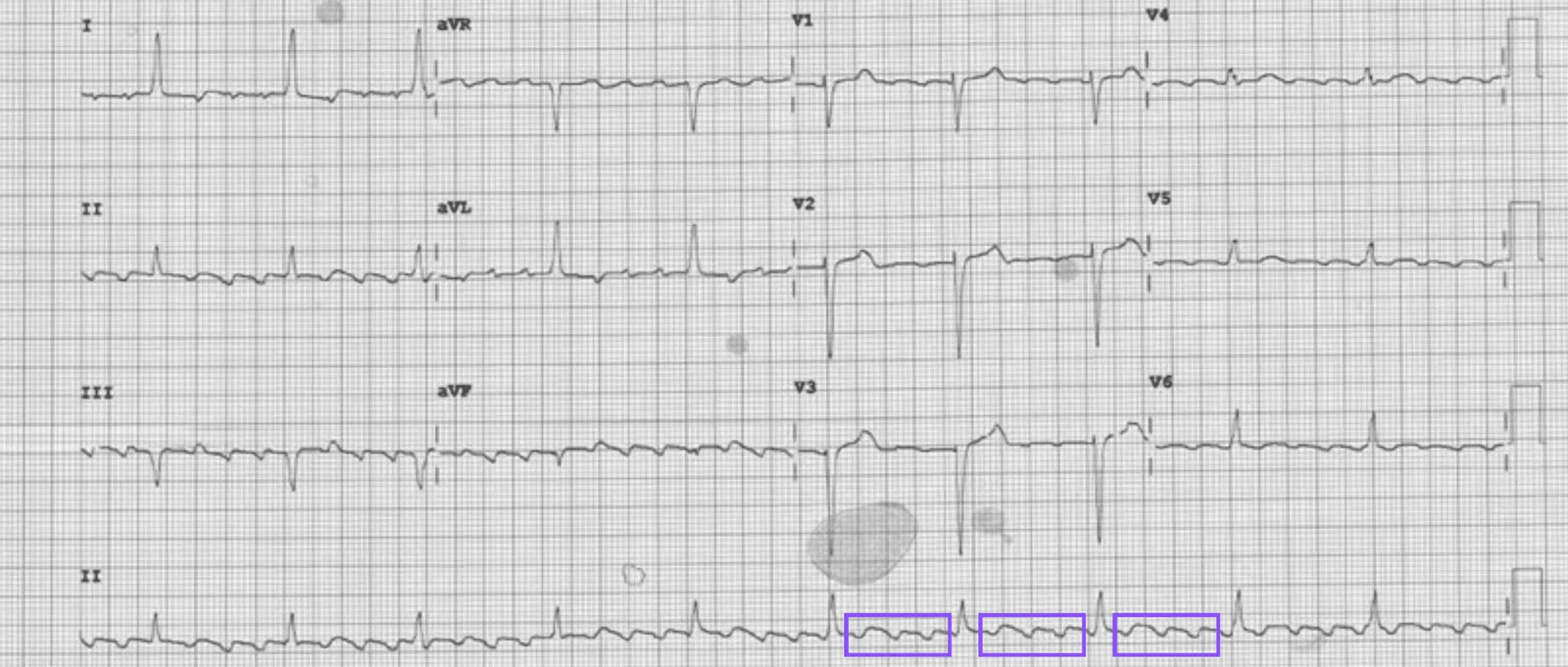
Twelve-lead ECG demonstrating typical atrial flutter with sawtooth flutter waves most prominent in the inferior leads and a 4:1 atrioventricular conduction pattern. The purple boxes highlight the characteristic “seesaw” pattern of flutter waves. ECG: electrocardiogram

A 12-lead electrocardiogram (ECG) obtained shortly after presentation demonstrated classic sawtooth flutter waves, most prominently visualized in the inferior leads (II, III, and aVF), consistent with typical atrial flutter (Figure 1). The organized atrial activity was regular and rapid, with an estimated atrial rate of approximately 240 to 250 beats per minute. There was a stable 4:1 atrioventricular conduction pattern, resulting in a controlled ventricular rate of approximately 60 beats per minute, which correlated with her relatively preserved hemodynamic status on presentation. The QRS complexes were narrow with normal duration, indicating intact intraventricular conduction without evidence of bundle branch block or pre-excitation. No acute ischemic ST-segment elevations or depressions were identified, and there were no reciprocal changes suggestive of myocardial ischemia. The corrected QT interval was mildly prolonged but remained below clinically high-risk thresholds for malignant ventricular arrhythmias.

**Figure 2:**
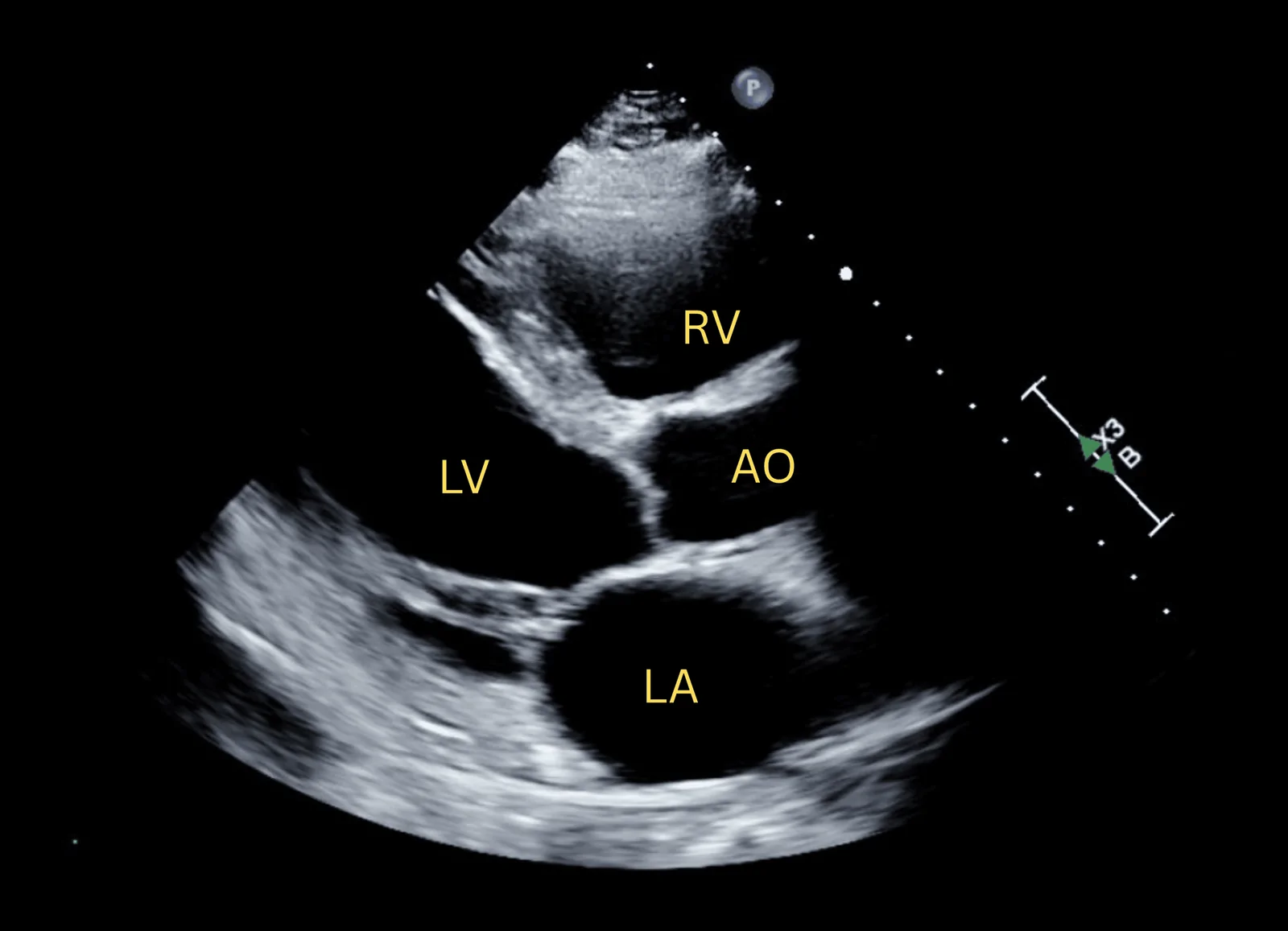
TTE demonstrating normal cardiac structure and function with preserved left ventricular ejection fraction (60-65%) and no significant valvular abnormalities. TTE: transthoracic echocardiogram; RV: right ventricle; LV: left ventricle; AO: aorta; LA: left atrium

A transthoracic echocardiogram (TTE) was subsequently performed to evaluate for underlying structural cardiac abnormalities as a potential contributor to the patient's atrial flutter (Figure 2). The study demonstrated preserved left ventricular systolic function with an estimated ejection fraction of 60-65%, normal left and right atrial dimensions, and normal right ventricular systolic function. No significant valvular abnormalities were identified, including the absence of clinically significant stenosis or regurgitation. There was no evidence of chamber enlargement, wall motion abnormalities, intracardiac thrombus, or other structural cardiac pathology.

Several alternative causes of atrial flutter were considered and evaluated. Myocarditis and acute myocardial ischemia were considered less likely given the absence of a recent infectious prodrome, normal high-sensitivity troponin I, lack of ischemic ECG changes, and subsequent echocardiographic demonstration of preserved ventricular function without regional wall motion abnormalities. Stimulant- or sympathomimetic-induced arrhythmia was considered unlikely based on the patient’s denial of illicit drug use, excessive caffeine or energy drink consumption, and exposure to stimulant or sympathomimetic medications. Thyrotoxicosis was excluded by a normal thyroid-stimulating hormone level. Structural and congenital cardiac abnormalities were also considered less likely given the absence of a personal or family history of congenital heart disease, sudden cardiac death, inherited arrhythmia syndromes, or cardiomyopathy, together with a normal TTE demonstrating preserved biventricular function, normal chamber dimensions, and no significant valvular or structural abnormalities. In the absence of these alternative precipitants, the profound hypomagnesemia, accompanied by hypokalemia and metabolic alkalosis in the setting of severe nutritional restriction and chronic stimulant laxative use, was considered the most likely reversible contributor to the atrial flutter.

Following identification of typical atrial flutter on ECG in the setting of profound hypomagnesemia, the patient was admitted to a telemetry-monitored medical floor for close cardiovascular observation and targeted electrolyte correction. Continuous cardiac monitoring was initiated upon arrival to the inpatient unit to assess for potential progression of arrhythmia, rapid ventricular response, or development of hemodynamic instability. Given that she remained hemodynamically stable with preserved blood pressure, intact mentation, adequate oxygenation, and a controlled ventricular response without clinical signs of acute decompensated heart failure, a conservative, etiology-directed rhythm strategy was pursued rather than immediate pharmacologic or electrical intervention. At the time of admission, the working clinical impression favored a reversible, metabolically mediated atrial arrhythmia resulting from significant electrolyte depletion in the setting of chronic stimulant laxative abuse and severe malnutrition associated with anorexia nervosa, rather than a primary structural or idiopathic cardiac conduction disorder.

Immediate management was directed toward rapid stabilization of the myocardial electrical environment through aggressive correction of identified electrolyte abnormalities. Intravenous (IV) magnesium sulfate was initiated promptly, beginning with a 4 g loading dose administered over approximately four hours, followed by a continuous infusion titrated based on serial serum magnesium measurements to maintain levels within the upper-normal physiologic range. In parallel, potassium repletion was initiated using both IV and oral potassium chloride supplementation, recognizing that hypokalemia is often refractory to correction in the setting of ongoing magnesium deficiency due to impaired cellular potassium retention. Serum electrolytes were closely monitored at frequent intervals, approximately every four to six hours during the initial treatment phase, to guide titration of replacement therapy, assess response, and prevent iatrogenic overcorrection. Serial laboratory assessments obtained over the first 24 hours of hospitalization demonstrated steady and progressive improvement in magnesium and potassium levels, along with gradual resolution of associated acid-base disturbances following electrolyte repletion (Table 2).

**Table 2:** Serial laboratory trends during electrolyte replacement demonstrating progressive correction of hypomagnesemia, hypokalemia, and associated metabolic abnormalities over the first 24 hours of hospitalization.

Laboratory Test	Admission (0 Hours)	6 Hours	12 Hours	24 Hours	Reference Range
Magnesium	0.8 mg/dL	1.4 mg/dL	1.8 mg/dL	2.0 mg/dL	1.7-2.4 mg/dL
Potassium	3.1 mmol/L	3.4 mmol/L	3.8 mmol/L	4.2 mmol/L	3.5-5.0 mmol/L
Calcium	8.4 mg/dL	8.6 mg/dL	8.7 mg/dL	8.8 mg/dL	8.5-10.5 mg/dL
Chloride	94 mmol/L	96 mmol/L	98 mmol/L	100 mmol/L	98-107 mmol/L
Bicarbonate	31 mmol/L	30 mmol/L	28 mmol/L	26 mmol/L	22-29 mmol/L

Continuous cardiac telemetry was maintained throughout the hospitalization to allow for real-time rhythm surveillance and detection of any clinically significant conduction abnormalities. During the initial phase of electrolyte replacement, the patient remained in atrial flutter with a stable ventricular response and no evidence of hemodynamic compromise. Throughout this period, no episodes of rapid ventricular response, significant pauses, or progression to higher-grade atrioventricular block were observed on continuous monitoring. Importantly, despite the persistence of atrial flutter on telemetry, the patient reported gradual and clinically meaningful symptomatic improvement within several hours of initiating magnesium sulfate therapy, including decreased frequency and intensity of palpitations as well as complete resolution of presyncopal symptoms and associated lightheadedness.

Given her overall stability, including the absence of hypotension, ischemic changes, or clinical signs of acute heart failure, pharmacologic rate-control therapy with beta-blockers or nondihydropyridine calcium channel blockers was intentionally deferred. This decision was made to avoid obscuring the intrinsic electrophysiologic response to electrolyte correction and to minimize the risk of iatrogenic bradycardia, particularly in the context of her baseline low-normal heart rate and suspected malnutrition-related autonomic dysregulation. Similarly, immediate electrical cardioversion was not pursued, as the arrhythmia was believed to be secondary to a reversible metabolic disturbance, and the duration of atrial flutter was considered to be less than 48 hours, thereby reducing concern for atrial thrombus formation and supporting an initial conservative management approach focused on correction of the underlying electrolyte abnormalities.

Approximately six to eight hours after initiation of IV magnesium repletion, continuous telemetry demonstrated abrupt spontaneous conversion from atrial flutter to normal sinus rhythm without any preceding ectopy, pauses, or administration of antiarrhythmic agents. The closest available serum magnesium measurement was 1.8 mg/dL at 12 hours, following progressive correction from an initial concentration of 0.8 mg/dL. At the time of rhythm conversion, the patient reported an immediate sensation described as a “release” or “settling” in the chest, followed by complete resolution of palpitations, dizziness, and lightheadedness. Cardiac monitoring following the event demonstrated normal sinus rhythm with complete resolution of flutter waves, no evidence of atrioventricular or intraventricular conduction delay, and normalization of the previously borderline QT interval.

Following stabilization of cardiac rhythm, electrolyte repletion was continued to maintain serum magnesium levels above 2.0 mg/dL and serum potassium levels above 4.0 mmol/L for an additional 24 to 48 hours to minimize the risk of early arrhythmia recurrence during physiologic recovery. Serial metabolic panels obtained during this period demonstrated continued correction and stabilization of serum magnesium and potassium concentrations, along with resolution of the associated metabolic alkalosis as intravascular volume status improved with isotonic fluid administration. No recurrence of atrial flutter or any other clinically significant arrhythmia was detected on continuous telemetry monitoring for the remainder of the hospitalization.

Once cardiovascular stability was achieved and sustained normal sinus rhythm was confirmed on continuous monitoring, clinical focus was shifted toward identification and management of the underlying drivers of the patient’s severe electrolyte derangements. A formal psychiatry consultation was obtained, which further characterized an active eating disorder consistent with anorexia nervosa, marked by severe and persistent restrictive eating patterns, significant cognitive distortion regarding body weight and shape, and ongoing compulsive use of stimulant laxatives as a maladaptive weight-control behavior. During the evaluation, the patient acknowledged a psychological reliance on laxatives as a perceived mechanism to prevent weight gain, despite understanding that this practice was medically harmful. She also expressed notable ambivalence toward complete cessation, reflecting the chronic and reinforcing nature of disordered eating behaviors. In addition, generalized anxiety disorder was identified as a significant comorbid condition contributing to heightened emotional distress, maladaptive coping strategies, and reduced adherence to previously prescribed psychiatric therapy.

Following psychiatric assessment, a structured, coordinated management plan was initiated involving internal medicine, cardiology, psychiatry, nutrition services, and social work to address both acute medical stabilization and long-term behavioral health needs. Given the patient’s severe chronic malnutrition and high risk for refeeding syndrome, nutritional rehabilitation was initiated cautiously with a carefully titrated caloric advancement strategy and close inpatient monitoring. Serial laboratory surveillance, including frequent assessment of phosphorus, magnesium, potassium, and serum glucose, was implemented to detect and manage early metabolic shifts associated with refeeding. Electrolyte supplementation was proactively incorporated into the nutritional plan to prevent recurrence of deficiencies during intracellular electrolyte redistribution. Behavioral health interventions emphasized motivational interviewing techniques aimed at enhancing treatment engagement, reducing laxative dependence, and improving insight into the physiologic consequences of disordered eating behaviors. Structured patient education sessions were also provided, focusing on the electrophysiologic effects of magnesium depletion, including increased atrial excitability, shortened refractory periods, and predisposition to reentrant tachyarrhythmias, as well as the potential for life-threatening cardiac events if left uncorrected. The relationship between chronic laxative use, gastrointestinal and renal electrolyte losses, and resultant cardiac instability was reviewed in detail to reinforce understanding of the reversible nature of her presenting arrhythmia.

By hospital day 3, the patient remained in sustained normal sinus rhythm without recurrence of palpitations, dizziness, or presyncopal episodes on continuous telemetry monitoring. Clinically, she demonstrated marked improvement in functional status, including increased energy levels, improved tolerance of oral intake under supervised nutritional guidance, and resolution of orthostatic symptoms that had been present on admission. Serial laboratory testing confirmed continued stability of serum electrolytes following transition from IV to oral supplementation, with no evidence of recurrent hypomagnesemia, hypokalemia, or acid-base derangements. No additional cardiac rhythm disturbances were observed for the remainder of her hospital course. At the time of discharge, the patient was clinically stable and was prescribed oral magnesium supplementation, short-term potassium replacement, and continuation of sertraline therapy. She was referred to an intensive outpatient eating disorder treatment program with integrated psychiatric and nutritional follow-up, and outpatient cardiology follow-up was arranged to monitor for recurrence of arrhythmia in the setting of metabolic recovery. At discharge, she demonstrated improved insight into the interplay between restrictive eating behaviors, laxative misuse, electrolyte disturbances, and cardiac manifestations, and expressed willingness to engage in ongoing structured treatment aimed at long-term recovery and relapse prevention.

## Discussion

A key feature of this case is the central role of hypomagnesemia in the development and maintenance of the atrial arrhythmia, particularly in the setting of coexisting electrolyte and acid-base abnormalities. Magnesium is a fundamental cofactor in cardiac electrophysiology, and its deficiency affects multiple ion channel systems simultaneously, including impairment of sodium-potassium ATPase activity, increased calcium influx through L-type calcium channels, and destabilization of myocardial resting membrane potential [6]. These effects collectively shorten atrial refractory periods and increase dispersion of refractoriness, thereby promoting reentrant circuits that can manifest as atrial flutter [3,4]. In this patient, the severity of hypomagnesemia likely created a highly arrhythmogenic substrate that was further amplified by concurrent hypokalemia, which itself impairs repolarization and enhances ectopic atrial activity. The observed metabolic alkalosis likely contributed additional electrophysiologic stress through extracellular volume contraction and intracellular potassium shifts, further lowering the threshold for arrhythmia initiation and maintenance [5]. Although hypokalemia is frequently implicated in arrhythmogenesis, the rapid resolution of rhythm disturbance following magnesium repletion in this case suggests that magnesium deficiency was the primary driver, with potassium imbalance acting as a contributing but secondary factor.

The clinical course provides important insight into the management of arrhythmias driven by correctable metabolic abnormalities and reinforces the importance of addressing the underlying cause rather than immediately targeting the rhythm itself. Despite the presence of sustained atrial flutter on admission, the patient remained hemodynamically stable with preserved blood pressure, adequate oxygenation, and a controlled ventricular response, which allowed for a conservative, physiology-driven management strategy. This stability permitted a deliberate decision to defer pharmacologic rate-control agents and electrical cardioversion in favor of correcting the underlying electrolyte disturbances. This approach was clinically meaningful, as it allowed the care team to observe the direct electrophysiologic response of the myocardium to restoration of electrolyte homeostasis without confounding effects from antiarrhythmic therapy. IV magnesium sulfate was appropriately selected as first-line therapy due to its dual role in correcting the measured deficiency and facilitating intracellular potassium repletion, which is often refractory when magnesium stores remain depleted [10]. The coordinated correction of both magnesium and potassium was essential, as isolated potassium supplementation alone would likely have been insufficient to restore electrical stability in this context [6,10].

The spontaneous conversion from atrial flutter to normal sinus rhythm within hours of magnesium sulfate administration is one of the most clinically significant observations in this case, as it provides a strong temporal association between correction of the electrolyte abnormalities and rhythm resolution. However, causality cannot be established definitively, as spontaneous conversion of atrial flutter cannot be completely excluded. The absence of antiarrhythmic agents or electrical cardioversion during this transition supports a temporal association between electrolyte correction and restoration of sinus rhythm, although spontaneous termination of atrial flutter remains a possible alternative explanation. The abrupt symptomatic improvement reported by the patient at the time of conversion further reinforces the clinical relevance of this electrophysiologic change. Continued telemetry monitoring demonstrated sustained maintenance of sinus rhythm throughout hospitalization, supporting the observation that rhythm stability coincided with correction of the metabolic abnormalities, although a causal relationship cannot be definitively established [2,3]. This pattern is particularly important in distinguishing potentially metabolically mediated atrial flutter from structurally mediated arrhythmias, which may demonstrate higher recurrence rates and often require long-term rhythm or rate-control strategies [4].

Another important aspect of this case is the identification of chronic stimulant laxative abuse as the underlying driver of the electrolyte disturbance and, ultimately, the arrhythmia. In patients with anorexia nervosa, laxative misuse represents a form of purging behavior that is often underreported and may be perceived by patients as a compensatory mechanism for caloric intake, despite having minimal effect on caloric absorption in the small intestine [9]. Chronic ingestion of stimulant laxatives can lead to repetitive gastrointestinal fluid losses, renal compensatory mechanisms that further deplete electrolytes, and progressive depletion of total body magnesium and potassium stores [11]. Over time, this results in a chronic state of volume depletion and electrolyte instability, as reflected in the patient’s metabolic alkalosis and hypochloremia [12]. These abnormalities not only predispose to arrhythmia but may also contribute to nonspecific symptoms such as fatigue, weakness, and orthostatic intolerance, which were present in this case but initially overshadowed by the more acute cardiac presentation [9]. This case therefore reinforces the importance of obtaining a detailed and nonjudgmental behavioral history in young patients with unexplained electrolyte abnormalities, as recognition of laxative abuse is essential for identifying the true etiology of otherwise unexplained arrhythmias.

Finally, the collaborative care approach was central to both acute stabilization and long-term risk reduction in this patient. While correction of electrolyte abnormalities resolved the immediate cardiac manifestation, the underlying risk for recurrence remained high in the absence of intervention targeting the eating disorder and associated psychiatric comorbidities [13]. Involvement of psychiatry was critical in identifying the severity of anorexia nervosa and the presence of maladaptive cognitive distortions regarding body image and weight control, as well as in addressing generalized anxiety disorder, which likely contributed to the reinforcement of restrictive and purging behaviors [14]. Nutritional services played an essential role in initiating a carefully monitored refeeding strategy to avoid refeeding syndrome, a known risk in severely malnourished individuals undergoing caloric restoration. Social work support further facilitated coordination of outpatient resources and continuity of care [15]. Without this integrated approach, there would be a substantial risk of recurrence of electrolyte disturbances and subsequent life-threatening arrhythmias, emphasizing that successful management of such cases requires both acute physiologic correction and sustained behavioral and nutritional intervention.

## Conclusions

This case illustrates that atrial flutter in young patients without structural heart disease may occur in the setting of severe, reversible metabolic disturbances. In this patient, chronic stimulant laxative use in the setting of anorexia nervosa was associated with profound hypomagnesemia, mild hypokalemia, and metabolic alkalosis, which likely contributed to the development of atrial flutter. The diagnosis was established through ECG and supported by laboratory findings, while other common potential causes of atrial flutter in young adults were evaluated and considered less likely based on the clinical history, laboratory testing, and cardiac imaging. Management focused on prompt correction of the underlying electrolyte abnormalities with IV magnesium sulfate and potassium repletion rather than immediate pharmacologic or electrical rhythm control, with spontaneous conversion to normal sinus rhythm occurring within hours and sustained rhythm stability thereafter. Although the temporal relationship between electrolyte correction and rhythm resolution supports a likely association, spontaneous conversion cannot be completely excluded, and causality cannot be established definitively from a single case. This case emphasizes the importance of recognizing electrolyte abnormalities as potentially reversible contributors to atrial arrhythmias and highlights the need for early identification of laxative misuse and eating disorders as potential underlying causes. In this patient, timely correction of the metabolic abnormalities, combined with coordinated interdisciplinary care addressing both medical and psychiatric contributors, was associated with rapid resolution of the arrhythmia and may have reduced the risk of recurrence and further complications. However, these findings are based on a single case and may not be generalizable to all patients with atrial flutter or electrolyte abnormalities.

## References

[1] Bagliani G, De Ponti R, Leonelli FM (2022). The history of atrial flutter electrophysiology, from entrainment to ablation: a 100-year experience in precision electrocardiology. Card Electrophysiol Clin.

[2] Graul EL, Nordon C, Rhodes K (2024). Factors associated with non-fatal heart failure and atrial fibrillation or flutter within the first 30 days post COPD exacerbation: a nested case-control study. BMC Pulm Med.

[3] Abdala-Lizarraga J, Quesada-Ocete J, Quesada-Ocete B, Jiménez-Bello J, Quesada A (2024). Cavotricuspid isthmus-dependent atrial flutter. Beyond simple linear ablation. Rev Cardiovasc Med.

[4] Whitehill R, Hill AC, Baskar S (2025). Thromboembolic complications from atrial fibrillation and atrial flutter in pediatrics and young adults: a multicenter study. J Cardiovasc Electrophysiol.

[5] Afzal MR, Savona S, Mohamed O, Mohamed-Osman A, Kalbfleisch SJ (2019). Hypertension and arrhythmias. Heart Fail Clin.

[6] Apell HJ, Hitzler T, Schreiber G (2017). Modulation of the Na,K-ATPase by magnesium ions. Biochemistry.

[7] Hansen BA, Bruserud Ø (2018). Hypomagnesemia in critically ill patients. J Intensive Care.

[8] Liu T, Li T, Xu D (2023). Small-conductance calcium-activated potassium channels in the heart: expression, regulation and pathological implications. Philos Trans R Soc Lond B Biol Sci.

[9] Nitsch A, Dlugosz H, Gibson D, Mehler PS (2021). Medical complications of bulimia nervosa. Cleve Clin J Med.

[10] Farahani M, Sharifinejad N, Hashemnejad M (2022). The effect of magnesium sulfate on maternal serum electrolytes and parathyroid hormone in pre-eclampsia. Int J Gynaecol Obstet.

[11] Sidler M, Mohebbi N, Hoorn EJ, Wagner CA (2019). Gut it out: laxative abuse mimicking distal renal tubular acidosis. Kidney Blood Press Res.

[12] Puckett L (2023). Renal and electrolyte complications in eating disorders: a comprehensive review. J Eat Disord.

[13] Kowalewska E, Bzowska M, Engel J, Lew-Starowicz M (2024). Comorbidity of binge eating disorder and other psychiatric disorders: a systematic review. BMC Psychiatry.

[14] Thompson CJ, Martin-Wagar CA (2025). Cognitive flexibility and emotion regulation in eating disorder patients with comorbid generalized anxiety and posttraumatic stress symptoms. Eat Disord.

[15] Krutkyte G, Wenk L, Odermatt J, Schuetz P, Stanga Z, Friedli N (2022). Refeeding syndrome: a critical reality in patients with chronic disease. Nutrients.

